# Is Transmural Healing an Achievable Goal in Inflammatory Bowel Disease?

**DOI:** 10.3390/ph18081126

**Published:** 2025-07-27

**Authors:** Ilaria Faggiani, Virginia Solitano, Ferdinando D’Amico, Tommaso Lorenzo Parigi, Alessandra Zilli, Federica Furfaro, Laurent Peyrin-Biroulet, Silvio Danese, Mariangela Allocca

**Affiliations:** 1 Department of Gastroenterology and Endoscopy, IRCCS San Raffaele Hospital and Vita-Salute San Raffaele University, 20132 Milan, Italy; faggiani.ilaria@hsr.it (I.F.); virginiasolitano@gmail.com (V.S.); damico.ferdinando@hsr.it (F.D.); parigi.tommaso@hsr.it (T.L.P.); zilli.alessandra@hsr.it (A.Z.); furfaro.federica@hsr.it (F.F.); sdanese@hotmail.com (S.D.); 2 Department of Epidemiology and Biostatistics, Western University, London, ON N6A 3K7, Canada; 3 Department of Gastroenterology, INFINY Institute, INSERM NGERE, CHRU Nancy, F-54500 Vandoeuvre-lès-Nancy, France; peyrinbiroulet@gmail.com

**Keywords:** computer tomography enterography, Crohn’s disease, inflammatory bowel disease, intestinal ultrasonography, magnetic resonance imaging, transmural healing, ulcerative colitis

## Abstract

**Background/Objectives**: In the era of treat-to-target strategies in inflammatory bowel disease (IBD), transmural healing (TH) is gaining recognition as a promising therapeutic goal. TH has been associated with significantly better long-term outcomes, including reduced rates of hospitalization, surgery, and the need for therapy escalation. Cross-sectional imaging techniques, such as intestinal ultrasound (IUS), magnetic resonance imaging (MRI), and computed tomography enterography (CTE), offer a comprehensive, non-invasive means to assess this deeper level of healing. This review explores how TH is currently defined across various imaging modalities and evaluates the feasibility and cost-effectiveness of achieving TH with available therapies. **Methods**: A literature search was conducted across PubMed, Scopus, and Embase using keywords, including “transmural healing”, “intestinal ultrasonography”, “magnetic resonance imaging”, “computed tomography enterography”, “Crohn’s disease”, “ulcerative colitis”, and “inflammatory bowel disease”. Only English-language studies were considered. **Results**: Despite growing interest, there is no standardized definition of TH across imaging platforms. Among the modalities, IUS emerges as the most feasible and cost-effective tool, owing to its accessibility, accuracy (sensitivity 62–95.2%, specificity 61.5–100%), and real-time capabilities, though it does have limitations. Current advanced therapies induce TH in roughly 20–40% of patients, with no consistent differences observed between biologics and small molecules. However, TH has only been evaluated as a formal endpoint in a single randomized controlled trial to date. **Conclusions:** A unified and validated definition of transmural healing is critically needed to harmonize research and guide clinical decision-making. While TH holds promise as a meaningful treatment target linked to improved outcomes, existing therapies often fall short of achieving complete transmural resolution. Further studies are essential to clarify its role and optimize strategies for deep healing in IBD.

## 1. Introduction

Crohn’s disease (CD) and ulcerative colitis (UC) are chronic inflammatory bowel diseases (IBDs) that significantly impact the quality of life of affected patients [1]. CD can involve any segment of the gastrointestinal tract, from the mouth to the anus, and affects all layers of the intestinal wall [2]. In contrast, UC typically affects the colon and rectum and has historically been considered a disease confined to the mucosa. However, recent evidence has highlighted the involvement of deeper intestinal layers, such as the submucosa [3,4].

In the era of advanced treatments for IBD, the introduction of biologic drugs and small-molecule therapies has shifted the therapeutic goal toward achieving deep disease control [5]. Until now, endoscopic healing has been the primary therapeutic target for IBD and remains, together with clinical remission, one of the key endpoints in randomized clinical trials (RCTs) for the approval of new drugs. The absence of macroscopic markers of inflammation, such as ulcers or erosions, during endoscopic assessment defines what is known as “mucosal healing” (MH) [6]. However, the achievement of mucosal healing should be considered an initial step in the resolution of gut inflammation in IBD rather than the ultimate therapeutic goal, given the transmural nature of the disease and the involvement of all bowel wall layers [6].

Within this context, the concept of transmural healing (TH) has gained increasing attention, as recognized in the STRIDE II consensus. Although TH is a challenging treatment target to achieve, it has been associated with reduced future corticosteroid use, fewer disease flares, and lower rates of hospitalization and surgery [6,7]. The definition of TH remains not fully clarified, particularly in UC, where the concept of transmural disease is relatively new. In fact, emerging evidence suggests that transmural alterations in UC represent a complex phenomenon, which, over time, may influence bowel motility and contribute to symptom persistence [8].

Cross-sectional imaging modalities play a pivotal role in the non-invasive assessment of transmural inflammation and healing. Intestinal ultrasonography (IUS), magnetic resonance imaging (MRI), and computed tomography enterography (CTE) have all demonstrated high diagnostic accuracy for detecting bowel wall thickening, edema, vascularity, and complications such as strictures and fistulas [7,9]. Among these, IUS and MRI are increasingly preferred due to their safety profiles, with IUS offering the added advantage of being real-time, bedside, repeatable, and cost-effective. MRI, on the other hand, provides excellent soft-tissue contrast and comprehensive assessment of deep tissue involvement and extramural complications, making it an essential tool in both clinical practice and research [10]. These imaging techniques allow for longitudinal monitoring of disease activity, including assessment of response to therapy, and have the potential to be integrated into treat-to-target strategies focused on achieving TH [11,12].

In parallel, the efficacy of available therapies in inducing TH is an emerging area of interest. While biologics and small molecules have demonstrated good efficacy in inducing mucosal healing, data regarding their impact on transmural outcomes remain limited and heterogeneous [13]. In addition, to date, no advanced therapy has demonstrated consistent superiority in achieving TH, and very few randomized controlled trials include TH as a predefined endpoint [12].

This review aims to explore the role of TH as an emerging treatment target, the utility of cross-sectional imaging in assessing TH, and the potential of advanced therapies to achieve this therapeutic goal.

## 2. Methods

We conducted a comprehensive search of the PubMed, Embase, and Scopus databases up until 31 December 2024, with the aim of identifying studies regarding transmural healing in inflammatory bowel disease. However, we ensured that our review included the latest relevant publications available up to 15 June 2025. To achieve this, we employed specific search terms such as “transmural healing”, “transmural response”, and “transmural remission”, in conjunction with “Crohn’s disease”, “ulcerative colitis”, “inflammatory bowel disease”, “CD”, “UC”, “IBD”, “intestinal ultrasound”, “magnetic resonance imaging”, “computed tomography enterography”, “IUS”, “MRI”, and “CTE”. We limited our search to articles published in the English language.

Our screening process involved two independent reviewers (I.F. and V.S.) who initially assessed titles and abstracts to identify potentially relevant studies. Subsequently, we examined the full texts of these selected articles to determine their eligibility for inclusion. Additionally, we manually scrutinized the reference lists of these articles to ensure that no relevant studies were overlooked during the electronic search. The final inclusion of abstracts and articles was based on their relevance to our research objectives.

## 3. Results

### 3.1. Definitions and Heterogeneity in Assessing Transmural Healing

#### 3.1.1. Variability in TH Definitions Across Studies and Guidelines

The definition of TH varies across studies and across different techniques (IUS, MRI and CTE), yet cross-sectional imaging remains the most valuable method for the evaluation of the disease from a different perspective [9,14].

Regarding IUS, early studies defined TH as normalization of bowel wall thickness (BWT) alone or BWT of ≤3 mm and a color Doppler signal (CDS) of 0 (no vascularization) or 1 (minimal vascular signals) [15,16,17,18]. An extended version included normalization of BWT and at least two additional parameters among CDS, bowel wall stratification (BWS), and absence of inflammatory fat (i-fat) [14]. Over time, the definition of TH has become more stringent, and recent studies have increasingly considered it as the normalization of all IUS parameters [12,19]. A recent expert consensus by the International Bowel Ultrasound (IBUS) group proposed a standardized definition of TH for both CD and UC. For CD, according to this consensus, TH is defined as a BWT of ≤3 mm with a normal CDS in both the small and large bowel. For UC, the definition of TH is a BWT of ≤3 mm with a normal CDS in the large bowel. However, in some patients, particularly those with diverticular disease, the sigmoid colon may exhibit an enlarged muscularis propria (outer hypoechoic layer), allowing for a BWT of up to 4 mm without indicating active inflammation. The consensus also provides recommendations on the timing of TH reassessment. In CD, TH should be reassessed between 26 and 52 weeks after treatment initiation, whereas in UC, an earlier reassessment at week 14 ± 2 is indicated [20].

The heterogeneity of available studies concerning MRI-based assessment in CD was clearly highlighted in a recent systematic review by Caron et al. Eighteen studies focusing on CD were included, with the Magnetic Resonance Index of Activity (MaRIA) score being the most commonly used tool to evaluate MRI remission [21,22]. However, the authors emphasize the inconsistency in the definitions of remission across studies. For example, MRI remission was variably defined as a MaRIA score < 7, a MaRIA score < 7 in all intestinal segments, or a total score < 11 across all segments in patients with baseline segmental scores ≥ 7 or ≥11 [21,23]. In other studies, remission was defined as a segmental MaRIA < 11 in areas with baseline scores ≥ 11, combined with a reduction of at least 5 points in those same segments. The review also underlines discrepancies in the timing of MRI assessments among studies [21]. In this context, the criteria proposed by Geyl et al. may serve as practical guidance for clinicians in routine care, defining MRI remission as a bowel wall thickness ≤ 3 mm, absence of contrast enhancement, and absence of complications (such as abscesses, strictures, or fistulae), with a median assessment time of 26 weeks after treatment initiation [24]. Of importance, one of the most recent studies on TH assessment via MRI by Revés et al. adopted a stricter definition of TH compared to earlier studies. Specifically, TH was defined as the complete normalization of all MRI parameters [25].

CTE is less commonly employed for disease assessment in IBD, and consequently, fewer studies are available compared to other imaging modalities. Moreover, the definition of TH on CTE varies considerably across the available literature. In a study by Deepak et al., TH was defined as the improvement of all assessed imaging parameters, including BWT, mucosal hyperenhancement, the presence of the comb sign, active inflammation, perienteric inflammation, fistulae, and strictures [26]. In contrast, Laterza et al. proposed a qualitative grading system in which an expert radiologist categorized three levels of remission—remission, mild to moderate remission, and moderate to severe remission—based on visual evaluation of BWT (≤3 mm), presence or absence of the target sign and comb sign, lymphadenopathy (<1 cm), sinus tracts, fibrofatty proliferation, perienteric stranding, and abdominal free fluid [27]. A more recent retrospective study adopted a simplified approach, defining TH as BWT ≤ 3 mm, normal mural signal intensity without mural hyperenhancement, and the absence of perienteric infiltration or penetrating complications [28].

#### 3.1.2. Challenges and Implications for Research and Clinical Practice

Currently, colonoscopy remains the gold standard for assessing MH in patients with IBD. However, it is an invasive procedure associated with significant costs and a considerable burden for patients [29]. In registration trials, endoscopic improvement/remission is often a primary endpoint, leading to the frequent use of colonoscopy, sometimes more than once per year. Despite its central role, patients generally prefer non-invasive modalities such as MRI, IUS, or CTE, particularly within tight-control monitoring strategies [30]. Rimola et al. recently demonstrated the feasibility of incorporating MRI into clinical trials for CD, as a complementary tool to endoscopy, particularly for a more accurate assessment of small bowel healing [31]. In contrast, a similar feasibility assessment for IUS in RCTs is currently lacking, yet IUS is recognized as represents a low-cost alternative compared to MRI, CTE, and endoscopy, while maintaining high diagnostic accuracy [32].

**Figure 1:**
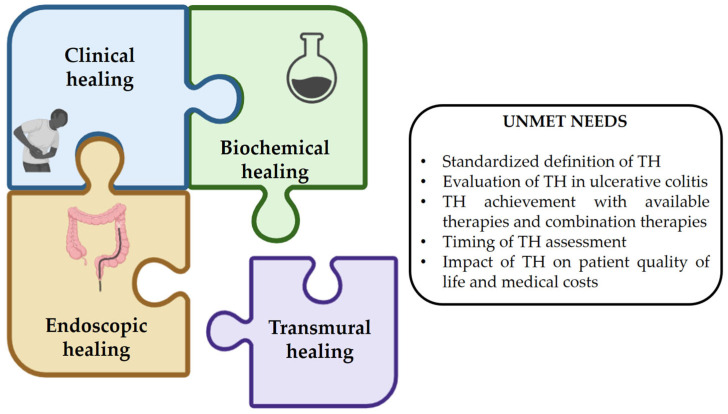
Transmural healing has not yet been established as a formal therapeutic target in the management of inflammatory bowel disease. This figure highlights the key data gaps and evidence needed to support the inclusion of transmural healing as a recognized treatment goal in clinical practice. TH: transmural healing.

In the routine management of patients with IBD, the ultimate goal is to improve long-term outcomes. In this context, several studies employing cross-sectional imaging have demonstrated that achieving TH is associated with better clinical outcomes than MH alone. In fact, Buisson et al. demonstrated that TH, as assessed by MRI, was predictive of sustained clinical corticosteroid-free remission (odds ratio [OR] 4.42, (95% CI 2.29–26.54); *p* = 0.042) and significantly reduced the risk of CD-related surgery (hazard ratio [HR] 0.16, (95% CI 0.043–0.63); *p* = 0.008) [33]. Interestingly, a study by Fernandes et al. found that patients who achieved TH on MRI had significantly lower rates of hospital admission (3%), therapeutic escalation (15.2%), and surgery (0%) compared to those with MH (*p* = 0.044; *p* = 0.027; *p* = 0.047, respectively) [34]. Similarly, Lafeuille et al. associated MRI healing with lower risk of bowel damage progression in patients with CD compared to endoscopic healing (HR = 0.09 [0.00–0.47], *p* = 0.005) [35]. When assessed using IUS, TH was associated with significantly better outcomes at one year, including a higher rate of steroid-free clinical remission (95.6%), lower hospitalization rates (8.8%), and no need for surgery (0%). In comparison, patients who achieved only MH had lower rates of steroid-free remission (75%), higher hospitalization rates (28.3%), and a 10% need for surgery. Those without any healing had markedly worse outcomes, with steroid-free remission in only 41%, hospitalization in 66.6%, and a 35.5% need for surgery [36]. (Figure 1).

### 3.2. Modalities for Assessing Transmural Healing

#### 3.2.1. Intestinal Ultrasonography

##### Sonographic Parameters of TH

IUS has gained increasing importance in the assessment of IBD activity over the past decade. This technique represents a non-invasive and cost-effective method for evaluating disease activity [8,37]. Validated IUS scoring systems, such as the International Bowel Ultrasound Segmental Activity Score (IBUS-SAS) and the Bowel Ultrasound Score (BUSS) for CD, as well as the Milan Ultrasound Criteria (MUC) for UC, are primarily based on two key parameters: BWT and CDS [38,39]. These scoring systems serve as reliable tools for assessing disease activity, alongside indirect signs of active disease, such as loss of the BWS, lymph node enlargement, and inflammatory mesenteric fat (i-fat) (Table 1).

- **Bowel wall thickness (BWT):** A BWT >3 mm has been shown to be more accurate than a 4 mm cut-off (88% sensitivity, 93% specificity vs. 75% sensitivity, 97% specificity, respectively) [40]. Notably, BWT measurement has a good interobserver agreement both in CD and UC (κ = 0.96 and κ = 0.63, respectively) [38,41].
- **Vascularization:** CDS has a moderate to good interobserver agreement (κ = 0.60 in CD and 0.83 in UC) [38,41].
- **Bowel wall stratification (BWS):** In active disease, BWS is often disrupted, with focal or extensive bowel wall alterations up to complete loss of stratification [42].
- **Lymph node enlargement:** Lymph nodes with a short-axis diameter >10 mm are more likely to be pathological. Interobserver reproducibility is good (κ = 0.61) [42].
- **Inflammatory mesenteric fat (i-fat):** When inflamed, mesenteric fat appears homogeneously hyperechoic. However, interobserver agreement for this parameter is fair to good (κ = 0.36–0.51) [38,41].

##### Accuracy, Reproducibility, and Clinical Evidence

A systematic review encompassing 179 studies evaluated the diagnostic accuracy of IUS, MRI, and CTE in inflammatory bowel disease (IBD). Specifically for IUS, 39 studies were analyzed. The pooled sensitivity of IUS for detecting disease activity ranged from 62% to 95.2%, while pooled specificity varied between 61.5% and 100%. Overall diagnostic accuracy was reported between 69% and 95% [43]. Regarding the diagnostic accuracy of IUS in different colorectal segments, the systematic review by Sagami et al. showed a diagnostic OR decreased from the right to the transverse and left colon and further to the rectum (diagnostic OR [95% CI] = 86.4 [19.8–376.8], 60.0 [13.9–259.1], 59.5 [14.0–252.5], 6.6 [1.4–32.1], respectively) [44]. Interestingly, IUS maintains its accuracy when compared to MRI and colonoscopy together in assessing localization (sensitivity 88%, specificity 96%), vascularization (sensitivity 87%, specificity 92%), and activity (sensitivity 92%, specificity 100%) [45].

One of the most commonly cited criticisms of IUS is its operator-dependent nature. However, interobserver agreement for IUS has been shown to be comparable to that of MRI, as demonstrated by study from the METRIC study investigators [46,47]. In this study, MRI scans from 73 patients were independently assessed by three radiologists. For newly diagnosed or relapsing patients, interobserver agreement for the presence of small bowel disease was 68% (κ = 0.36) and 78% (κ = 0.56), respectively. Agreement for disease extent was lower, at 43% (κ = 0.14) for new diagnoses and 53% (κ = 0.07) for relapsing cases. Notably, agreement was highest in cases involving multisegment disease, lesions greater than 5 cm in length, mural thickness exceeding 6 mm, and increased mural T2 signal intensity [46].

**Table 1:** Intestinal ultrasonography and magnetic resonance imaging scoring system for disease activity assessment in IBD.

Score Name	Key Parameters Included	Formula
**Intestinal Ultrasound (IUS) Scores**		
IBUS-SAS (International Bowel Ultrasound Segmental Activity Score) [38]	BWT, CDS, BWS, inflammatory mesenteric fat	4 × BWT + 15 × i-fat + 7 × CDS + 4 × BWS
BUSS (Bowel Ultrasound Score) [39]	BWT, CDS	0.75 × BWT + 1.65 × BWF, where BWF = 1 if present, or BWF = 0 if absent
MUC (Milan Ultrasound Criteria) [48]	BWT, CDS	1.4 × BWT + 2 × BWF, where BWF = 1 if present, or BWF = 0 if absent
**Magnetic Resonance Imaging (MRI) Scores**		
MaRIA (Magnetic Resonance Index of Activity) [49]	BWT, RCE, edema, ulcers	= 1.5 × wall thickness + 0.02 × RCE + 5 × edema + 10 × ulceration
Simplified MaRIA [50]	BWT, edema, fat stranding, ulcers	(1 × thickness > 3 mm) + (1 × edema) + (1 × fat stranding) + (1 × ulcers)

BWT: bowel wall thickness; CDS: color Doppler signal; BWS: bowel wall stratification; RCE: relative contrast enhancement.

#### 3.2.2. Magnetic Resonance Imaging (MRI)

##### MRI Markers of TH

MRI scores and markers for assessing disease activity are shown in Table 1. BWT, relative contrast enhancement (RCE), presence of edema, and presence of ulcers are the MRI parameters included in the MaRIA score, which is the most widely used index for evaluating CD activity [49,51]. Below are the most frequently utilized MRI parameters to evaluate TH:

- **Edema**: High signal intensity on T2-weighted or STIR images due to increased water content in inflamed bowel walls. Qualitative assessment with no standard quantitative threshold but often graded based on signal intensity relative to nearby muscle or unaffected bowel.
- **Bowel Wall Thickening**: The cut-off of >3 mm is commonly used as abnormal; can also be evaluated as a continuous variable with higher values indicating more severe disease.
- **Increased Contrast Enhancement**: Detected post-gadolinium contrast administration on T1-weighted sequences. A relative enhancement >50% compared to pre-contrast images is often indicative of active inflammation, though exact thresholds may vary.
- **Diffusion-Weighted Hyperintensity**: High signal on DWI images (especially at high b-values like b800–1000 s/mm^2^). It is a qualitative evaluation of the presence of hyperintensity and supports active inflammation.
- **Apparent Diffusion Coefficient (ADC)**: Quantitative measure of water molecule diffusion and inversely related to disease activity. ADC < 1.3 × 10^−3^ mm^2^/s has been proposed in some studies to differentiate active from inactive disease, but cut-offs vary.
- **Injected Sequences (RCE)**: Quantifies the degree of bowel wall enhancement after contrast administration. The formula used to assess it is RCE = [(Signal intensity post-contrast – pre-contrast)/pre-contrast signal] × 100%. RCE > 100% is often considered indicative of active inflammation in Crohn’s disease, though this threshold can vary between institutions.

##### Sensitivity and Specificity Compared to Other Techniques

Rate of sensitivity and specificity for MRI are variable in the literature. On the other hand, the METRIC study is the largest prospective study comparing MRI with IUS [48]. Sensitivity and specificity rates for MRI vary across the literature. However, the METRIC study remains the largest prospective trial directly comparing MRI with IUS. The study found that the sensitivity of MRE for detecting small bowel disease extent was significantly higher than that of IUS (80% [95% CI, 72–86] vs. 70% [95% CI, 62–78]; *p* = 0.027). Similarly, MRE demonstrated greater sensitivity for detecting disease activity (97% 95% CI, [91–99]) compared to IUS (92% [95% CI, 84–96]; *p* = 0.025). The specificity of MRE for small bowel disease extent was also significantly higher than that of IUS (95% [95% CI, 85–98] vs. 81% [95% CI, 64–91]; *p* = 0.039). In contrast, for the presence of small bowel disease, specificity was 96% (95% CI, 86–99) with MRE and 84% [95% CI, 65–94] with IUS, a difference that did not reach statistical significance (*p* = 0.054) [48].

#### 3.2.3. Computed Tomography Enterography (CTE)

CTE is not so used in the clinical practice as MRI and IUS, yet estimated sensitivity and specificity of the three imaging modalities are high, without a significant difference among them [52]. CTE is widely accessible and offers good reproducibility in image quality with the added benefit of being quick to perform. However, like MRI and unlike IUS, CTE requires the use of both oral and intravenous contrast agents and must be interpreted by a radiologist. In contrast, IUS can be performed at the bedside by a trained gastroenterologist, providing a significant advantage by enabling real-time clinical decision-making and timely treatment adjustments [53]. However, the major drawback of CTE compared to IUS and MRI is its reliance on ionizing radiation. This is a particularly relevant concern in younger patients and in the context of chronic conditions such as IBD, where repeated imaging over time may be necessary [53].

### 3.3. Is Transmural Healing Achievable with Current IBD Therapies?

#### 3.3.1. Evidence from Biologics and Small Molecules in CD

The rate of TH achievement can vary significantly depending on the definition used (Table 2 and Table 3). Overall, TH remains a challenging therapeutic goal. Reported rates of TH achievement range between 20% and 40%, whereas MH rates in clinical trials can reach up to 60% in UC [54,55]. In contrast, MH rates in CD tend to be lower, averaging around 45% [56]. To date, only a few RCTs have included TH as an endpoint. Among them, the DIVERGENCE 1 trial—a phase 2 study evaluating filgotinib in the treatment of small bowel Crohn’s disease—incorporates transmural assessment as a secondary endpoint. [23]. TH was assessed using MRI, with a predefined threshold of MaRIA < 7 in all small bowel segments. At week 24, radiological healing was achieved in 6.3% and 8.0% of patients receiving filgotinib 100 mg and 200 mg, respectively [23]. The STRIDENT RCT tried to assess TH in a specific population involving patients with symptomatic stenosing CD [57]. Patients (n = 77) were randomly assigned to receive adalimumab randomized to high-dose adalimumab plus thiopurine (68%) or standard adalimumab monotherapy (33%). Both IUS and MRI were used to evaluate stenosis. On IUS, stricture resolution was defined by normalized BWT (<3 mm), absence of hyperemia, and normalization of prestenotic dilatation. On MRI, resolution required a normal BWT (≤3 mm), normal luminal diameter, and prestenotic dilatation <3 cm. At 12 months, stenosis resolution was achieved in 29% of patients on IUS and 22% on MRI, with no differences between treatment groups [57].

No significant difference in the rate of reaching TH among therapies was observed [12]. Even in the pediatric population, the HEAL study demonstrated no significant difference in TH achievement between therapies [58]. This prospective, longitudinal observational study evaluated MRI response in children receiving maintenance therapy with either adalimumab (n = 26) or immunomodulators (n = 36). TH was defined using the Pediatric Inflammatory Crohn’s MRE Index (PICMI) score, with a threshold of ≤10. Although a decrease in PICMI score (>20) without therapy change was more frequently observed in the adalimumab group compared to the immunomodulator group (54% vs. 31%, *p* = 0.01), normalization of MRI findings remained rare in both groups (9% vs. 6%, *p* = 0.62) [58]. Notably, only in the study by Calabrese et al. did ustekinumab demonstrate lower rates of TH achievement, with a higher proportion of patients showing unchanged or worsened lesions compared to those treated with anti-TNF agents. (infliximab vs. ustekinumab: HR = 2.7; 95% CI, 1.9–6.4; *p* = 0.017; adalimumab vs. ustekinumab: HR = 2.1; 95% CI, 1.12–3.9; *p* = 0.02) [19]. In addition, achieving TH might require a longer duration of treatment. This is exemplified by a progressively decreasing number needing treatment over time following initiation of advanced therapy: 6.1 at 3 months compared to 3.6 at 12 months [12]. Supporting this, the sub-study of the STARDUST trial assessing TH with IUS demonstrated a gradual increase in TH rates over the course of treatment: 1.8% at week 4, 6.3% at week 8, 11.9% at week 16, and 24.1% at week 48 [59]. Similarly, in the sub-study of the VERSIFY trial, radiologic healing (MaRIA score of <7 in all bowel segments) was observed in 21.9% and 38.1% of patients treated with vedolizumab at week 26 and 52, respectively [31].

#### 3.3.2. Evidence from Biologics and Small Molecules in UC

In UC, data on TH remain limited [60]. However, emerging evidence suggests its potential prognostic value. A recent study demonstrated that IUS-based transmural assessment was a stronger predictor of colectomy risk than endoscopic evaluation [61]. A BWT of >6 mm was considered an optimal cutoff for predicting the risk of colectomy in the first 3 months after diagnosis (OR 38, 95% CI, 8–270, *p* < 0.001) [62].

In light of these results, the importance of TH in UC is becoming increasingly evident. Madsen et al. demonstrated the feasibility of achieving TH in UC, reporting even higher rates than those typically observed in CD. In their prospective study of 139 UC patients, TH was defined as normalization of all IUS parameters. Remarkably, 59% (82/140) of patients achieved TH within 3 months of diagnosis, and this rate increased to 75% (95/126) after one year [62].

Furthermore, a recent abstract presented by *Lim et al.* at the ECCO Congress in 2025 provided additional support for the clinical relevance of TH in UC. The study demonstrated that patients achieving TH experienced significantly improved outcomes compared to those with endoscopic healing alone. Relapse-free survival was the primary endpoint: the risk of relapse at one and two years was 7.5% and 20.5%, respectively, in the TH group, versus 29.4% and 64.3% in the non-TH group (*p* = 0.004) [63].

**Table 2:** Rates of transmural healing achievement with currently available therapies in Crohn’s disease. TH: transmural healing; Anti-TNFα: anti-tumor necrosis factor α; IUS: intestinal ultrasound; BWT: bowel wall thickness; CD: Crohn’s disease; IFX: infliximab; ADA: adalimumab; MH: mucosal healing; MRI: magnetic resonance imaging; NH: non-healing; CTE: computer tomography enterography; VDZ: vedolizumab; ASUC: acute severe ulcerative colitis; RCT: randomized control trial; CDS: color Doppler signal; BWS: bowel wall stratification; i-fat: inflammatory fat; IBD: inflammatory bowel disease; and MaRIA: Magnetic Resonance Index of Activity.

Therapy	Author, Ref.	Study Design	Imaging	Definition of TH	N	Intervention	Results
**Anti-TNF** **α**	Paredes, 2010 [64]	Prospective, longitudinal	IUS	BWT: 3 mm, absence of a color Doppler signal, absence of intestinal complications (fistula, abscess)	24	1 year of treatment with IFX or ADA	TH: 5/17 (29.0%)
	Castiglione, 2013 [15]	Prospective, longitudinal	IUS	BWT ≤ 3 mm	133	2 years of treatment with IFX or ADA	TH: 17/66 (25.0%) MH: 25/66 (38.0%)
	Moreno, 2014 [17]	Prospective, longitudinal	IUS	BWT: 3 mm, color Doppler signal (using Limberg score 0–1), % of parietal enhancement increase less than 46%	30	1 year of treatment with IFX or ADA (±ISS)	TH: 15 (83.3%) MH: 18 (60%)
	Ripollés, 2016 [18]	Prospective, multicenter	IUS	BWT: 3 mm, color Doppler signal (using Limberg score 0), absence of intestinal complications (fissures, fistulas, inflammatory masses)	51	1 year of treatment with IFX or ADA	TH week 12: 7/51 (14.0%) TH week 52: 15/51 (29.5%)
	Castiglione, 2017 [16]	Prospective, longitudinal	IUS, MRI	BWT ≤ 3 mm	40	2 years of treatment with IFX or ADA	TH (IUS): 10/40 (25.0%) TH (MRI): 9/40 (23.0%) MH: 14/40 (35.0%)
	Orlando, 2018 [65]	Prospective, longitudinal	IUS	BWT ≤ 3 mm	30	52 weeks of treatment with IFX or ADA	TH week 14: 8/30 (27.0%) TH week 52: 9/30 (30.0%)
	Castiglione, 2019 [38]	Prospective, single center, longitudinal	IUS	Normalization of BWT of all inflamed segments involved in CD; BWT ≤ 3	218	2 years of treatment with IFX or ADA	TH: 68 (31.2%) MH: 60 (27.5%) NH: 90 (41.3%)
	Paredes, 2019 [66]	Prospective, single center	IUS	Normalization of BWT (<3), Limberg 0 or 1	33	1 year of treatment with IFX or ADA	TH: 14 (42.2%) NH: 19 (57.6%)
	Bossuyt, 2021 [67]	Prospective	MRI	MaRIA score < 7 in all segments	36	52 weeks of treatment with IFX	TH: 30.3% MH: 71.0%
	Lafeuille, 2021 [35]	Retrospective, database review	MRI	TH: MH+ MRI healing MRI healing: no MRI signs of inflammation and no complication (stricture, abscess, or fistula)	154	IFX	TH: 5/28 (17.9%) MRI healing: 6/30 (20.0%) NH: 14/80 (17.5%)
						ADA	TH: 4/28 (14.3%) MRI healing: 2/30 (6.7%) NH: 16/80 (20.0%)
	Calabrese, 2022 [12]	Prospective, multicenter	IUS	Normalization of all parameters	188	1 year of treatment with IFX, 31 patients (16%)	TH: 37.0% NH: 23.0%
						1 year of treatment with ADA, 103 patients (55%)	TH: 26.5% NH: 33.0%
	Oh, 2022 [68]	Retrospective, single center	MRI, CTE	BWT < 3 mm, absence of mural hyperenhancement, normal mural signal, no perienteric infiltration, absence of newly developed or worsening preexisting stricturing or penetrating complications	392	Anti-TNF therapy for more than one year	MH+TH: 114/392 (29.1%) TH: 41/392 (10.4%) MH: 59/392 (15.0%) NH: 178/392 (45.4%)
	Revés, 2025 [25]	Multicenter, retrospective	MRI	Normalization of all MRI parameters	154	IFX	TH: 20/85 (23.5%) NH: 65/85 (76.5%)
						ADA	TH: 8/55 (14.5%) NH: 47/55 (85.5%)
**Vedolizumab**	Lafeuille, 2021 [35]	Retrospective, database review	MRI	TH: MH+ MRI healing MRI healing: no MRI signs of inflammation and no complication (stricture, abscess or fistula)	154	VDZ	TH: 0/28 (0.0%) MRI healing: 1/30 (3.3%) NH: 3/80 (3.7%)
	Calabrese, 2022 [12]	Prospective, multicenter	IUS	Normalization of all parameters	188	1 year of treatment with VDZ, 24 patients (13%)	TH: 27.2% NH: 41.0%
	Rimola, 2024 [31]	Prospective, multicenter	MRI	MaRIA score of <7 in all bowel segments	59	26 and 52 weeks of treatment with VDZ	TH week 26: 8/37 (21.9%) TH week 52: 4/22 (38.1%)
	Carter, 2025 [69]	Prospective, multicenter	IUS		70	6 months of treatment with VDZ	TH: 9/28 (32.1%)
	Revés, 2025 [25]	Multicenter, retrospective	MRI	Normalization of all MRI parameters	154	VDZ	TH: 0/1 (0.0%) NH: 1/1 (100%)
**Ustekinumab**	Lafeuille, 2021 [35]	Retrospective, database review	MRI	TH: MH+ MRI healing MRI healing: no MRI signs of inflammation and no complication (stricture, abscess, or fistula)	154	ustekinumab	TH: 0/28 (0.0%) MRI healing: 0/30 (0.0%%) NH: 3/80 (3.7%)
	Miranda, 2021 [70]	Prospective	MRI/IUS	MRI: complete healing of all layers of the bowel wall IUS: TH ≤ BWT 3 mm and normal IUS examination	92	ustekinumab	TH: 15/75 (20.0%) TH: 11/75 (14.7%) MH: 26/75 (34.0%)
	Calabrese, 2022 [12]	Prospective, multicenter	IUS	Normalization of all parameters	188	1 year of treatment with ustekinumab, 30 patients (16%)	TH: 20.0% NH: 48.0%
	Kucharzik, 2023 [59]	RCT phase 3b	IUS	Normalization of BWT, CDS, BWS, and i-fat	77	24 weeks of treatment with ustekinumab	TH: (13/54) 24.1%
	Revés, 2025 [25]	Multicenter, retrospective	MRI	Normalization of all MRI parameters	154	VDZ	TH: 4/13 (30.8%) NH: 9/13 (69.2%)
**Upadacitinib**	Bezzio, 2024 [71]	Observational, cohort	IUS	BWT < 3 mm	64	upadacitibib	TH: 15/52 (28.8%)
**Filgotinib**	D’Haens, 2023 [23]	Randomized control trial, phase 2	MRI	MaRIA < 7 in all segments	78	filgotinib for 24 weeks	TH filgotinib 100 mg: 2/32 (6.3%) TH filgotinib 200 mg: 2/25 (8.0%)

**Table 3:** Rates of transmural healing achievement in ulcerative colitis when a clear definition of transmural healing was applied. TH: transmural healing; IUS: intestinal ultrasound; BWT: bowel wall thickness; IFX: infliximab; ADA: adalimumab; CDS: color Doppler signal; BWS: bowel wall stratification; i-fat: inflammatory fat; MRI: magnetic resonance imaging; CTE: computer tomography enterography; and ASUC: acute severe ulcerative colitis.

Author	Study Design	Imaging	Definition of TH	N	Intervention	Results
Maaser, 2019 [72]	Sub-analysis of TRUST&UC, a prospective, observational, multicenter study	IUS	BWT < 4 mm for sigmoid colon and <3 mm for the descending colon	224	ADA, IFX, or golimumab	TH week 6: 21/44 (47.7%)
Helwig, 2022 [32]	Post-hoc analysis of TRUST studies	IUS	Complete TH: BWT, CDS, i-fat normalized	171	-	TH: 45.0%
Saleh, 2023 [73]	Retrospective study	IUS	BWT < 3 mm + normalization of all parameters (CDS ≤ 1, i-fat, BWS)	39	Mesalamine, biologics, small molecules	TH: 14/39 (35.9%)
Gilmore, 2023 [74]	Case series	IUS	BWT < 3 mm in the most affected segment with a modified Limberg score of 0	6	ASUC treated with upadacitinib	TH week 8: 4/6 (66.7%)
Lim, 2024 [63]	Prospective, single center, IBD registry study	MRI, CTE	Absence of inflammation on either CT or MR enterography	51	ustekinumab	TH: 16.7%
Madsen, 2025 [62]	Prospective, multicenter, population-based, inception, cohort study	IUS	If no inflammation was present (normal BWT, CDS, BWS, i-fat)	193	-	TH week 3: 82/140 (59.0%) TH 12 months: 95/126 (75.0%)

#### 3.3.3. Factors Influencing the Likelihood of Achieving TH

Several factors may influence the likelihood of achieving TH in patients with CD.

Shorter disease duration (less than 24 months) has consistently been associated with higher rates of TH. In the study by Castiglione et al., disease duration was identified as a predictor of non-healing (OR 3.03; 95% CI, 1.15–7.94; *p* = 0.02) [16]. Similarly, Maconi et al. reported a significant inverse association between disease duration and TH achievement (−0.94 [−1.75 to −0.13], *p* = 0.02) [75]. In the same study, higher baseline BWT was also associated with a lower probability of achieving TH (95% CI, 0.11–1.21; *p* = 0.02) [75]. These findings are consistent with earlier data from Calabrese et al., where greater BWT at baseline significantly predicted lower TH rates at 3 months (OR 0.69; 95% CI, 0.5–0.94; *p* = 0.018) and at 12 months (OR 0.65; 95% CI, 0.48–0.89; *p* = 0.006) [20].

Results from a pediatric population (≤18 years of age) including 98 patients with CD or UC showed a positive correlation between body mass index (BMI) and BWT. This multicenter, retrospective, cohort study demonstrated that both univariate (unadjusted β = 0.004 [95% CI = 0.001–0.008], *p* = 0.014) and multivariable (adjusted β = 0.006 [95% CI = 0.001–0.011], *p* = 0.038) analysis confirmed a significant association between BWT and weight [76]. However, findings from an adult population revealed that in multivariate analysis higher BMIs were associated with a greater likelihood of achieving TH (CI 95% 0.02 to 0.33; *p* = 0.01) [75].

A study by Ripollés et al. further highlighted the influence of disease phenotype, showing that 42% of patients without transmural complications achieved TH, compared to only 5% of those with fistulizing or stricturing disease [18]. Similar results were observed by Maconi et al., who found, in a univariate analysis of 68 CD patients treated with anti-TNF agents, that an inflammatory (non-fibrotic) phenotype was significantly associated with TH (59% vs. 15%; *p* = 0.002) [75].

Disease localization also appears to impact TH rates. Higher healing rates have been observed in proximal small bowel segments compared to the terminal ileum: 33.3% in the jejunum, 10% in the distal ileum, and 4.5% in the terminal ileum. Similarly, colonic disease showed higher TH rates than ileal disease (50.0% [8/16] vs. 13.2% [5/38], respectively) [31,59].

Importantly, early initiation of biologic therapy also seems to favor TH. A recent multicenter, retrospective study investigated the impact of timing of advance therapy initiation on TH achievement [25]. Among the 154 patients with CD included, 38% (59 patients) began biological therapy within 12 months and 47% (73 patients) started within 24 months of diagnosis. Starting treatment within 12 months of diagnosis was associated with significantly higher TH rates compared to later initiation (32% vs. 14%, *p* < 0.01; adjusted OR 3.2, 95% CI 1.4–7.7, *p* < 0.01) [25]. Furthermore, TH was independently linked to a reduced risk of bowel damage progression (HR 0.25; 95% CI, 0.09–0.70; *p* = 0.008), a lower likelihood of requiring CD-intestinal surgery (HR 0.20; 95% CI, 0.05–0.81; *p* = 0.025), and decreased need for therapy escalation (HR 0.35; 95% CI, 0.14–0.89; *p* = 0.03)) [25].

#### 3.3.4. Ongoing Clinical Trials Evaluating TH as an Endpoint

There are currently three studies with transmural healing as the endpoint:

- **TRENCH 1 (TRansmural hEaliNg Definition in Crohn’s Disease; NCT05903066)**: This is a multicenter, prospective, observational, cross-sectional study involving patients with CD with an indication for MRE based on routine clinical practice. The primary objective is to define the depth or grading of TH based on specific radiologic features observed during MRE. The study aims to standardize the radiologic interpretation of TH in CD, providing a more structured and reproducible definition for future clinical applications and research.
- **REASON (Transmural Healing and Disease-Modifying Effect of Guselkumab in Crohn’s Disease Patients; NCT06408935)**: This is a phase 3b, open-label, multicenter Study. Participants receive guselkumab high dose (200 mg subcutaneous (SC) every 4 weeks or low dose (100 mg SC every 8 weeks). The study aims to evaluate the efficacy of guselkumab in achieving TH using MaRIA score with an evaluation at week 48.
- **VECTORS—A Study to Evaluate Transmural Healing as a Treatment Target in Crohn’s Disease (NCT06257706)**: This phase 4 trial aims to evaluate whether a treat-to-target strategy that includes corticosteroid-free (CS-free) IUS outcomes in addition to clinical symptoms and biomarkers is superior to a strategy based solely on clinical symptoms and biomarkers in achieving CS-free endoscopic remission, as measured by the SES-CD.

## 4. Discussion

TH in not yet a formal target in IBD; however, it has emerged as a promising goal, particularly in CD [5]. To fully understand the clinical implications of TH, it is essential to first establish a clear and standardized definition across imaging modalities, including IUS, MRI, and CTE, to enable comparability across studies. The most recent consensus on the definition of TH dates back to 2022 [20]. Since then, a substantial amount of new literature has emerged, and novel scoring systems have been validated for assessing disease activity. While bowel wall thickening remains the most consistently used parameter across published studies, an updated and harmonized definition of TH is urgently needed. Our expectation is the development of a clear and unambiguous definition of TH. For this reason, the establishment of an international expert consensus has become a pressing priority.

Defining specific thresholds for TH is also of paramount importance. The DEVISE-CD project by Buisson et al. led to the development of the first validated MRI-based scoring system for assessing TH [77]. The score, referred to as the “C-score”, is applied to actively inflamed segments, identified by the presence of mural edema, and incorporates three parameters: bowel wall thickness (as a continuous quantitative measure), the presence of ulcers, and the presence of enlarged lymph nodes. To date, the C-score remains the only validated imaging score specifically designed to quantify transmural disease activity and healing in CD [77].

The optimal cross-sectional imaging modality for assessing TH remains a matter of debate. While MRI, IUS, and CTE have demonstrated moderate to good agreement with endoscopic findings, each has distinct advantages [45]. Rimola et al. have shown the feasibility of incorporating MRI into clinical trials, particularly for assessing small bowel disease, where IUS views may be suboptimal [31]. On the other hand, IUS has generated robust evidence of its utility in monitoring TH and is preferred by patients due to its non-invasive nature, low cost, and suitability for tight disease monitoring [30,73].

What is increasingly clear is that TH is associated with superior long-term outcomes, especially when combined with MH [68]. A comprehensive approach that incorporates both TH and MH may represent the optimal therapeutic target in IBD management. Several studies have demonstrated that patients achieving TH experience fewer disease flares, lower corticosteroid dependency, and reduced rates of hospitalization and surgery [34,35,77]. Ongoing trials such as VECTORS (NCT06257706), REASON (NCT06408935) and TRENCH1 (NCT05903066) may further elucidate the role of TH as a treatment target in CD.

It is important to note that current data predominantly pertain to CD. The clinical relevance of TH in UC—traditionally viewed as a mucosal disease—is still in its early stages and requires further investigation [8]. In particular, key questions that need to be addressed include the ability of IUS to accurately assess TH in the rectum, the challenge of differentiating transmural changes due to fibrosis from those related to active inflammation and the functional consequences of fibrosis [78].

Due to the lack of a standardized definition, reported TH achievement rates vary widely, generally ranging from 20% to 40%. To date, no advanced therapy has demonstrated clear superiority in achieving TH [12]. However, early initiation of treatment and longer treatment duration have both been associated with higher rates of TH. At the moment, only one RCT has included TH as a defined endpoint, and only two sub-studies of RCTs are currently available to provide higher-quality data on this outcome [23,31,59].

An unresolved issue is the optimal timing of TH assessment during follow-up. Recent expert consensus suggests monitoring at weeks 26 and 52 in CD, and at week 12 in UC, following therapy initiation [20]. Nevertheless, data from the STARDUST sub-study showed that changes in BWT could be detected as early as week 4 and that a lack of early transmural response was predictive of persistent endoscopic activity at week 48 [59]. These findings support the potential for earlier therapeutic optimization based on IUS findings; however, further evidence is needed to substantiate this approach.

Finally, the cost-effectiveness of targeting TH remains uncertain. Pursuing TH as a routine therapeutic endpoint could entail significant financial implications for healthcare systems. Therefore, comprehensive cost-effectiveness analyses are critically needed to evaluate whether the improved outcomes associated with TH justify the potential increase in healthcare resource utilization. Nonetheless, previous studies using the Markov model have already demonstrated that tight control strategies in IBD management can lead to a reduction in hospitalization rates and absenteeism-related costs [79].

Looking ahead, artificial intelligence is poised to play a transformative role in the assessment of IBD. Emerging studies on radiomic models demonstrate promising potential to support automated detection of disease activity, thereby enhancing the standardization of IUS, MRI and CTE interpretation across institutions [80]. Initial results demonstrated sensitivity and specificity rates of up to 93.8%, along with strong agreement between expert radiologists and the AI model (κ = 0.76, 95% CI 0.73–0.80) [80,81]. Additionally, AI use was associated with faster image interpretation [81].These advancements may also contribute to more consistent assessment of key concepts such as TH [51].

## 5. Conclusions

TH represents a potential future therapeutic target in the management of IBD. Its assessment through cross-sectional imaging modalities—such as IUS, MRI, and CTE—may reduce the reliance on invasive procedures like endoscopy. Emerging evidence suggests that TH is associated with improved clinical outcomes, including a lower incidence of disease flares, reduced corticosteroid dependence, and decreased rates of hospitalization and surgery when compared to MH. These findings provide a strong rationale for considering TH as a formal treatment endpoint.

Among the available imaging techniques, IUS is particularly promising as a cost-effective, non-invasive tool that can be performed in real time by trained gastroenterologists, potentially enhancing decision-making during routine clinical practice. However, the widespread adoption of TH as a clinical endpoint is currently limited by the lack of a standardized definition across imaging modalities. Establishing uniform criteria and thresholds for TH is essential. Additionally, while most existing data pertain to CD, further investigation is needed to explore the relevance and utility of TH in UC, where the transmural nature of inflammation has been historically underestimated. Future research should prioritize prospective validation of TH as a treatment target across diverse populations, including those with UC, and explore its integration into treat-to-target algorithms.

## Data Availability

No new data were generated or analyzed in support of this research.

## Abbreviations

The following abbreviations are used in this manuscript:

ADA	adalimuamab
BWT	bowel wall thickness
BWS	bowel wall stratification
CD	Crohn’s disease
CDS	color Doppler signal
CS-free	corticosteroid-free
CTE	computed tomography enterography
DH	deep healing
EH	endoscopic healing
HR	hazard ratio
IBD	inflammatory bowel disease
IFX	infliximab
IUS	intestinal ultrasound
MaRIA	Magnetic Resonance Index of Activity
MH	mucosal healing
MRI	magnetic resonance imaging
NH	non-healing
OR	odds ratio
RCT	randomized controlled trial
RH	radiologic healing
SES-CD	Simple Endoscopic Score for Crohn’s Disease
TH	transmural healing
PICMI	Pediatric Inflammatory Crohn’s MRE Index
UC	ulcerative colitis
VDZ	vedolizumab
